# Babesiosis and Malaria in the United States: Epidemiology, Research Funding, Medical Progress, & Recommendations for Improvement

**DOI:** 10.3390/epidemiologia6040076

**Published:** 2025-11-06

**Authors:** Ryan P. Jajosky, Wenhui Li, Audrey N. Jajosky, Philip G. Jajosky, Sean R. Stowell

**Affiliations:** 1 Joint Program in Transfusion Medicine, Brigham and Women’s Hospital, Harvard Medical School, 630E New Research Building, 77 Avenue Louis Pasteur, Boston, MA 02115, USA; weli21@bwh.harvard.edu (W.L.);; 2 Biconcavity Inc., Lilburn, GA 30047, USA; jajosky@gmail.com; 3 Department of Pathology and Laboratory Medicine, University of Rochester Medical Center, Rochester, NY 14586, USA

**Keywords:** US public health, global health, healthcare economics, foreign aid, NIH, CDC, National Center for Emerging and Zoonotic Infectious Diseases (NCEZID), research priorities, disease burden, underfunding, emerging infectious diseases, neglected diseases, rare diseases, orphan diseases, morbidity and mortality, parasitology, hematology

## Abstract

Background: Babesiosis and malaria are infectious diseases caused by the intraerythrocytic parasites *Babesia* and *Plasmodium*, respectively. While no human red blood cell (RBC) receptors have been shown to be essential for *B. microti* (*Bm*) invasion, Duffy (ACKR1) was reported to be essential for *P. knowlesi* and *P. vivax* invasion in 1975 and 1976, respectively. This suggests additional medical progress is needed for babesiosis, warranting a detailed analysis. Methods: Given similarities in the target cell of infection, data about babesiosis and malaria cases in the US were obtained from the Centers for Disease Control and Prevention (CDC). Research funding was quantified using National Institutes of Health (NIH) data, and medical progress was evaluated through a literature review. Results: Over the 5-year span of 2018–22, there were 9799 and 7722 confirmed babesiosis and malaria cases, respectively. Confirmed babesiosis cases exceeded malaria cases in 4 of 5 years. In 2022, babesiosis and malaria data were either not reported or unavailable to the CDC by ten and one US state(s), respectively. Regarding babesiosis, it is likely that the vast majority of cases were due to domestically acquired *Bm*, in the context of no chemoprophylaxis. Concerning malaria, >90% of US cases were imported from foreign locations, ~95% of cases were linked with not taking chemoprophylaxis, *and P. falciparum* (*Pf*) was the most common cause. From 2018–22, babesiosis and malaria were the underlying cause of death for 70 and 32 US residents, respectively. NIH funding estimates suggest ~$4 million in support of babesiosis and ~$169 million for malaria in 2024. There are many malaria-inspired medications, two malaria vaccines, and hundreds of characterized *Plasmodium* proteins, while these measures of medical progress are far behind for babesiosis. Outside of the US, there are >200 million malaria cases per year, while babesiosis is rare. Conclusions: In the US from 2018–22, there were more babesiosis cases and deaths than malaria. Decades of robust CDC and NIH funding for malaria led to its elimination from the US, improved medical knowledge and interventions, and reduced foreign morbidity and mortality. These data suggest that leveraging similar approaches used for malaria, including increased NIH and CDC funding for babesiosis, would likely lead to progress (e.g., improved treatment). Babesiosis qualifies as both a rare and an orphan disease.

## 1. Introduction

The United States (US) has the highest annual number of cases of human babesiosis, a sometimes-fatal parasitic infectious disease [1]. In 2011, the US Centers for Disease Control and Prevention (CDC) made babesiosis a nationally notifiable condition [2,3]. Because of increasing cases [2,3] and the emergence of drug-resistant parasites [4], there is a need to better understand babesiosis to improve public health and medical care.

Babesiosis and malaria are similar in that they are both caused by intraerythrocytic protozoan parasites called *Babesia* and *Plasmodium*, respectively [5,6]. Both are single-celled organisms that have an apical complex for host cell invasion and are categorized in the Apicomplexa phylum and the Aconoidasida class. *Babesia* can be pear-shaped and are in the Piroplasmida order [7,8,9]. *Plasmodium* produce haemozoin pigment when detoxifying heme from hemoglobin metabolism, are pleomorphic, and are in the Haemosporida order. Piroplasmida and Haemosporida have been called “sister groups” [8].

Babesiosis is endemic in the US, with most human cases occurring in the Northeast, some in the upper Midwest, and fewer cases in the Northwest and elsewhere. While malaria is not endemic in the US, it is much more common worldwide, where it represents one of the greatest disease burdens to humans. Malaria is perhaps the most significant infectious disease to shape human evolution.

In the US, *Babesia* is mostly transmitted to humans by deer ticks (*Ixodes scapularis*) and rarely by blood transfusion and other routes. In contrast, *Plasmodium* parasites are predominantly spread to humans by mosquitoes that feed on *Plasmodium*-infected humans. Babesiosis is primarily a zoonotic disease, while malaria caused by *P. falciparum* (*Pf*), the species that causes most deaths, is not zoonotic. Both babesiosis and malaria can cause flu-like symptoms. Parasitized red blood cells (RBCs) can be sticky (e.g., cytoadherence, rosetting), cause microvascular obstruction, partly due to parasitic molecules on the RBC surface [10], and can hemolyze. This can lead to anemia, jaundice, weakness, and other signs and symptoms, such as organ damage, neurological complications, and death. 

The pathogens that cause babesiosis and Lyme disease are primarily transmitted by *Ixodes scapularis* ticks [11]. In the US, *B. microti* (*Bm*) is the major cause of babesiosis, while *Borrelia burgdorferi* is the bacterium that causes most cases of Lyme disease. For both pathogens, mice, including the white-footed mouse (*Peromyscus leucopus*), are key mammalian hosts. Thus, there is a similar geographic distribution of both infectious diseases.

As transfusion medicine physicians in Boston, Massachusetts (in the Northeastern US), we are aware of the risk of RBC-transfusion-transmitted *Babesia* and *Plasmodium* and are occasionally consulted about RBC exchange transfusion for treatment of these diseases. We recognize the need to advance medical care for both babesiosis and malaria patients and have published numerous articles about both diseases. It is our impression that despite the US having the most human babesiosis cases [1], there is very little knowledge or investigation regarding this infectious disease. For a more objective assessment, we compared human babesiosis to malaria in the US simply because they are both caused by intraerythrocytic parasites. Although these diseases differ significantly in global impact, we also evaluated the potential role of National Institutes of Health (NIH) funding in driving medical progress, with the hope that the progress achieved in malaria research could be applied to improve public health and medical care regarding babesiosis.

## 2. Methods

### 2.1. Epidemiologic Data

Data regarding babesiosis and malaria cases in the US were obtained from CDC WONDER (Wide-ranging ONline Data for Epidemiologic Research) [12] and then clicking on the National Notifiable Diseases Surveillance System (NNDSS) Annual Summary Data Query. In the request form, the “select demographic” option was changed to either “regions/states”, “states”, “month”, “age”, “sex”, “race”, or “ethnicity”. For the “disease” filter, either “malaria”, “babesiosis, total”, “babesiosis, confirmed”, or “babesiosis, probable” was selected. Incidence data was obtained by checking the box for “Rates” in section 1 of the request form and leaving the default (calculate rate per 100,000) in section 3. For the “year” filter, 2018 through 2022 was selected. Data for all 50 US states, the District of Columbia (D.C.), New York City (NYC), and sometimes US territories were exported and analyzed. Because the CDC reports data for New York state, excluding NYC, and NYC as separate entities, these two values were added to obtain the numbers for New York state.

The current babesiosis case definition on the CDC website is from 2025 [13], but the 2011 definition was the prior version and applicable to this study [14]. The CDC WONDER database lists three options for babesiosis, which are total, confirmed, or probable cases. Total cases are equal to the sum of confirmed and probable cases. Confirmed cases have confirmatory test results and ≥1 objective or subjective clinical evidence criteria. To briefly summarize, laboratory confirmatory tests can involve polymerase chain reaction (PCR), nucleic acid amplification, animal inoculation, or light microscopy of stained blood smears, while laboratory-supportive tests rely on immunoglobulin levels against *Babesia*. CDC notes that it can be difficult to distinguish *Babesia* and *Plasmodium* on blood smears.

The current malaria case definition on the CDC website is from 2014 [15]. It lists case classifications as confirmed or suspected. Confirmed cases are based on microscopic examination of blood films or nucleic acid testing. According to the NNDSS annual event code list Excel file, the print criteria for malaria are confirmed cases, meaning that CDC WONDER shows only confirmed malaria cases (not suspected) [16].

Data about the underlying cause of death were obtained from CDC’s WONDER by clicking on “2018–2023: Underlying cause of death by single-race categories”, and data from 2018–22 were exported. All 50 US states and the D.C. were included. The year 2023 was excluded to match the timespan of available case data (2018–22). Under the heading “group results by”, “cause of death” was selected. Under “select a cause of death”, the ICD-10 code >B60.0 (babesiosis) was picked. For malaria deaths, all subgroups under >B50 (*Plasmodium falciparum* malaria), >B51 (*Plasmodium vivax* malaria), >B52 (*Plasmodium malariae* malaria), >B53 (other parasitologically confirmed malaria), >B54 (unspecified malaria), >P37.3 (congenital falciparum malaria), and >P37.4 (other congenital malaria) were selected. Regarding ICD-10 codes about “complications of medical and surgical care”, the categories of >Y41.2 (antimalarials and drugs acting on other blood protozoa) and >Y41.8 (other specified systemic anti-infectives and antiparasitics) were excluded because they do not distinguish whether the person had babesiosis, malaria, or neither. For example, antiparasitics such as quinine can be used to treat both babesiosis and malaria. Data from 2018–22 were exported. Sometimes, the results were grouped by the following: census region, sex, Hispanic origin, ten-year age groups, and single race 6 (which means 6 race categories, including American Indian or Alaska Native, Asian, Black or African American, other or multi-race, Native Hawaiian or other Pacific Islander, and white).

Data about multiple causes of death were obtained from CDC’s WONDER by clicking on “multiple cause of death (final)”. The option “2018–2023” was selected. Under the heading “group results by”, “multiple cause of death” was selected. The methods used in the prior paragraph were followed, except that the ICD-10 codes regarding poisoning by anti-infectives and antiparasitics (>T37.2 and >T37.8) were excluded because they do not distinguish whether the person had babesiosis, malaria, or neither.

### 2.2. NIH Funding Estimates

NIH funding by Research, Condition, and Disease Categories (RCDC) was used to determine the NIH’s estimate of funding allocated to malaria, tickborne diseases, Lyme disease, and other diseases [17]. Of note, 2021 was the first year in which tickborne disease funding was reported.

Because babesiosis is not part of the RCDC list, NIH RePORTER was used to estimate babesiosis and malaria funding [18]. The “fiscal year” was set to 2023 or 2024. The “agency/institute/center” was set to NIH. For babesiosis, under “text search”, the terms “babesia” and “babesiosis” were entered with “OR” logic, and the search was limited to the project title. For malaria, under “text search”, the terms “malaria”, “plasmodia”, and “plasmodium” were entered with “OR” logic, and the search was limited to the project title. Although these words may have been in “project terms” or the “abstract”, only projects that had these terms in the title were included. The data were exported to an Excel file.

The NIH specifies, “Please note that if the hit list contains both a subproject and its parent grant, the subproject funding is already included in the parent project funding amount.” To address this, the name of the parent grant for each subproject was looked up. If the parent grant was in the search results, then the subproject funding was excluded. If the parent grant was not in the search results, then the subproject funding was included. 

### 2.3. Bm Articles on PubMed

To determine how many PubMed articles about *Bm* were published in 2024, the search query (“Babesia microti”[Title]) AND ((“1 January 2024”[Date—Publication]: “31 December 2024”[Date—Publication])) was submitted. This looked for articles published in 2024, with the exact term “Babesia microti” in the article’s title. The country affiliation of the last author was used to assign the nation where the article came from.

## 3. Results

### 3.1. Recent US Public Health Efforts

Differences exist regarding laboratory and genomic support provided by CDC for babesiosis and malaria. For example, CDC requests that clinical laboratories send them pre-treatment blood samples from malaria patients for species confirmation, providing the results free of charge [19]. In addition, CDC tests *Plasmodium* for drug resistance [19]. These efforts have been incredibly important in monitoring and reducing malaria, and similar opportunities could be offered for babesiosis.

CDC also performs rapid genetic sequencing of *Plasmodium* during malaria outbreaks, while equivalent efforts have not been made for babesiosis. Notably, >150 cases of malaria acquired within the US have occurred in the past 50 years [20]. In 2023, nine *P. vivax* infections were acquired in the US, prompting the CDC to immediately develop sequencing strategies, which successfully traced parasite origins and transmission patterns. Given the risk of malaria becoming endemic in the US again, this is understandable. Similarly, CDC’s National Center for Emerging and Zoonotic Infectious Diseases (NCEZID) Strategic Plan for 2018–2025 explicitly mentions support for malaria, even in Africa, but does not mention babesiosis [21].

Babesiosis and malaria are nationally notifiable diseases in the US, which means that states voluntarily report case data to the CDC [22]. States are not required to report. Concerning malaria, only South Carolina case data were unavailable in 2022. Regarding babesiosis, ten states did not report data: Alaska, Colorado, Georgia, Hawaii, Idaho, Mississippi, Nevada, New Mexico, Oklahoma, and Pennsylvania. Strikingly, Pennsylvania is known to have babesiosis cases [23] and borders New York, which typically has the most cases per year. Because clinicians tend to be more aware of malaria, babesiosis may be disproportionately underdiagnosed and underreported. Ultimately, US public health efforts are more advanced for malaria than for babesiosis.

### 3.2. Cases in the US

**Figure 1:**
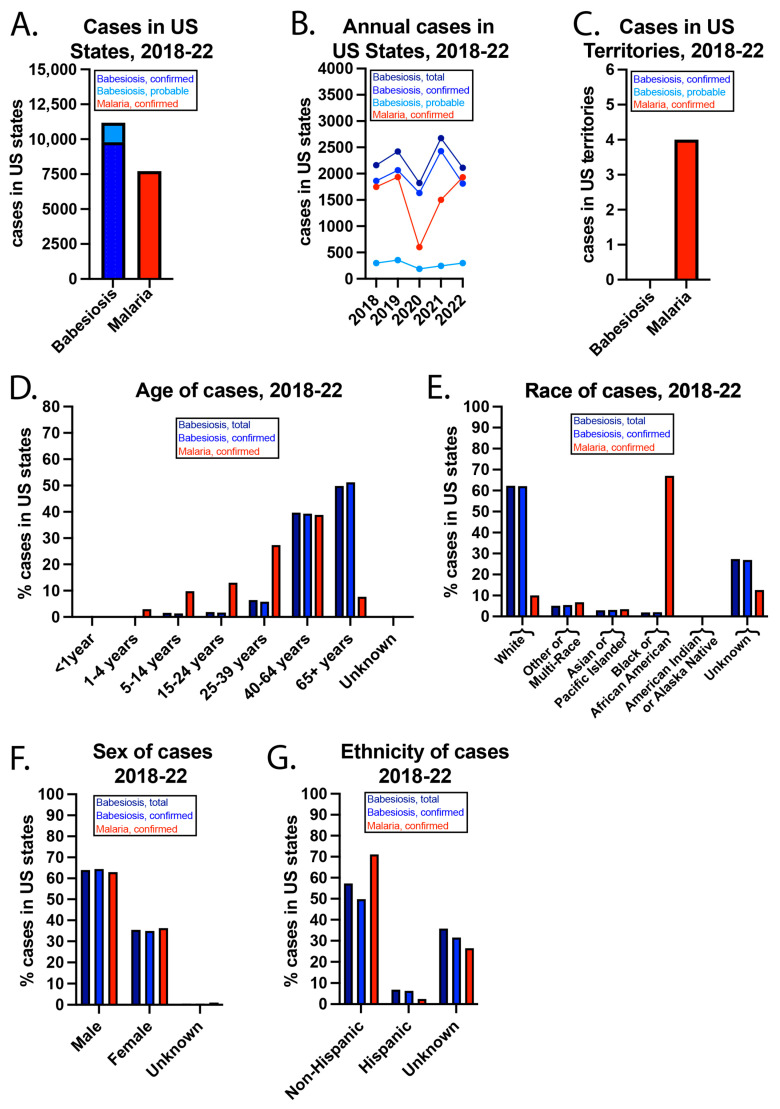
US babesiosis and malaria case details from 2018–22. (**A**) Babesiosis and malaria cases in US states (including D.C.) during the 5-year period and (**B**) annually. (**C**) Cases in US territories. (**D**) Age, (**E**) race, (**F**) sex, and (**G**) ethnicity of the cases from 2018–22.

From 2018–22, there were 11,185 total babesiosis cases (confirmed + probable), 9799 confirmed babesiosis cases, and 7722 confirmed malaria cases (Figure 1). The WONDER database has the following note: “Cases are assigned to the reporting jurisdiction submitting the case to NNDSS if the case’s country of usual residence is the United States, a U.S. territory, unknown, or the country is not reported; otherwise, the case is assigned to the non-U.S. residents category, beginning with cases reported in year 2020.” [24]. Only in the year 2022 were confirmed babesiosis cases fewer than confirmed malaria cases. From 2018–22 in US territories, there were 0 and 4 total babesiosis and confirmed malaria cases, respectively.

The COVID-19 pandemic impacted case data from 2019–22. CDC notes specific geographic locations where there may be incomplete data reporting due to the pandemic [24]. The pandemic also led to travel restrictions, and nearly all malaria cases in the US are “imported” due to foreign travel. For example, the CDC states that 93.5% of malaria cases in 2022 were imported, for 6.3% of cases, the person was lost to follow-up, <0.1% were due to blood exposure, and for 0.15% the cause was unknown [25]. Of the imported cases in 2022, >90.2% were associated with travel to Africa, most often to visit friends and relatives. Around 95% of cases were associated with not taking the chemoprophylaxis. *Pf* was found in 84.5% of the samples in which the species was determined.

While babesiosis cases were most common in individuals ≥65 years, malaria cases peaked in persons 40–64. It is well known that babesiosis is more severe in the elderly, immunocompromised persons, and individuals without a functioning spleen [2]. It is unclear why there are so few children with malaria, as this group develops the most severe disease [26]. Regarding race, >60% of babesiosis cases were in whites, while more than 60% of malaria cases were in blacks or African Americans. CDC has reported that US babesiosis cases are most common in whites [2], likely reflecting demographic differences in certain New England states such as Maine, New Hampshire, and Vermont, where *Babesia* infections are relatively common compared to other areas of the US. In contrast, the higher prevalence of malaria in blacks or African Americans is thought to be related to travel to areas where *Plasmodium* infections are endemic, such as Sub-Saharan Africa. More than 60% of both babesiosis and malaria cases were in men. Non-Hispanics accounted for almost 60% of total babesiosis cases, nearly 50% of confirmed babesiosis cases, and >70% of malaria cases. For a large percentage of cases, ethnicity was unknown.

**Figure 2:**
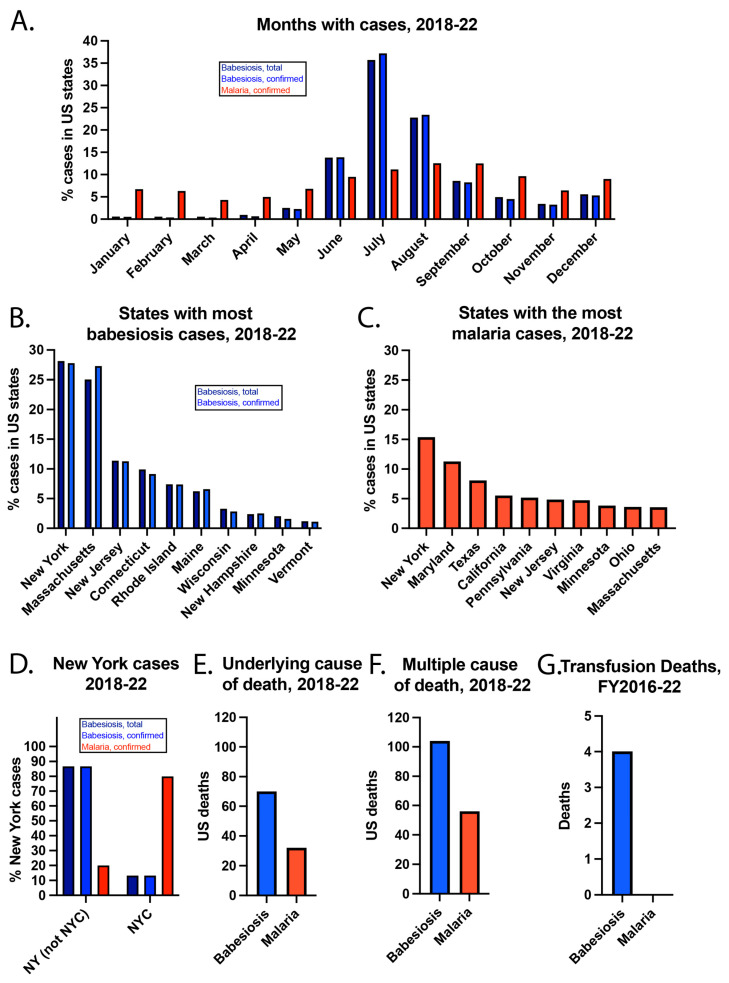
Additional details about US babesiosis and malaria cases from 2018–22. (**A**) Months of the cases, (**B**) states with the most babesiosis cases, (**C**) states with the most malaria cases, and (**D**) New York cases. Babesiosis and malaria reported as the (**E**) underlying cause of death or listed amongst (**F**) multiple causes of death. (**G**) Transfusion deaths from FY2016–22.

While babesiosis cases peaked in July, malaria cases peaked in August (Figure 2). In addition, most babesiosis cases were in June, July, and August, which corresponds to when *I. scapularis* nymph ticks are most active in seeking a blood meal [27]. In contrast, malaria cases were more evenly spread throughout the year. Babesiosis cases were most concentrated in the Northeast and Midwest, with more than 50% of cases occurring in New York and Massachusetts. Malaria cases were more evenly spread over the US. New York had the most babesiosis and malaria cases. While >70% of New York babesiosis cases were outside of New York City (NYC), >70% of New York malaria cases were in NYC.

The incidence of confirmed babesiosis was highest in Maine (12.78/100,000), Rhode Island (9.69/100,000), and Vermont (7.88/100,000) in the year 2022. The highest incidence states are in the Northeast, where most babesiosis cases occur. The incidence of confirmed malaria was highest in Maryland (3.78/100,000), NYC (2.77/100,000), and D.C. (1.64/100,000) in the year 2022. While heavily populated cities tend to concentrate infectious diseases [28], local transmission of malaria in the US is extremely rare. Thus, the concentration of malaria cases in NYC (the headquarters of the United Nations), the US capital and nearby Maryland may be due to a high proportion of politicians, ambassadors, and military personnel in these areas who travel internationally.

### 3.3. Deaths of US Residents

From 2018–22, babesiosis and malaria were the underlying causes of death for 70 and 32 US residents, respectively. Babesiosis and malaria were listed among multiple causes of death for 104 and 56 individuals, respectively. This means that babesiosis or malaria was one of multiple factors that contributed to a person’s death. There is additional information about the individuals who died of babesiosis or malaria, but the CDC “suppresses” some data, meaning it will not show the actual values when numbers are low. This is done to protect the privacy of the cases and their families. For example, CDC’s data use restrictions mention that deaths of nine or fewer persons cannot be reported or presented [29]. According to CDC policy, numbers are not typically reported if simple arithmetic can reveal the exact value of suppressed numbers >0. Thus, a deliberately incomplete description of deaths is provided.

When babesiosis was the underlying cause of death, 58/70 (82.9%) persons were residents of the Northeast Census Region, which includes Connecticut, Maine, Massachusetts, New Hampshire, New Jersey, New York, Pennsylvania, Rhode Island, and Vermont. Also, 42/70 (60%) persons were male, and 64/70 (91.4%) were white. In terms of ages, 19/70 (27.1%) were 65–74 years old, 19/70 (27.1%) were 75–84 years old, and 16/70 (22.9%) were ≥85 years old.

When malaria was the underlying cause of death, 15/32 (46.9%) persons were residents of the South Census Region, which includes Alabama, Arkansas, Delaware, D.C., Florida, Georgia, Kentucky, Louisiana, Maryland, Mississippi, North Carolina, Oklahoma, South Carolina, Tennessee, Texas, Virginia, and West Virginia. Also, 21/32 (65.6%) of persons were male, and 16/32 (50%) were black or African American, while 16/32 (50%) were white.

According to the Food and Drug Administration (FDA), there were 4 deaths due to *Bm* being transmitted by transfusion from fiscal year (FY) 2016–2020, but none for *Plasmodium* [30]. In FY 2021–2022, there were no deaths reported to the FDA that were linked with either parasite being transmitted by transfusion. This is likely because the FDA approved nucleic acid testing (NAT) for *Babesia* detection in blood donors in 2018 [31]. This was followed by large blood collection organizations, such as the American Red Cross, using this testing for all donations in locations specified in the FDA guidance [32].

### 3.4. Funding Estimates and Published Articles

**Figure 3:**
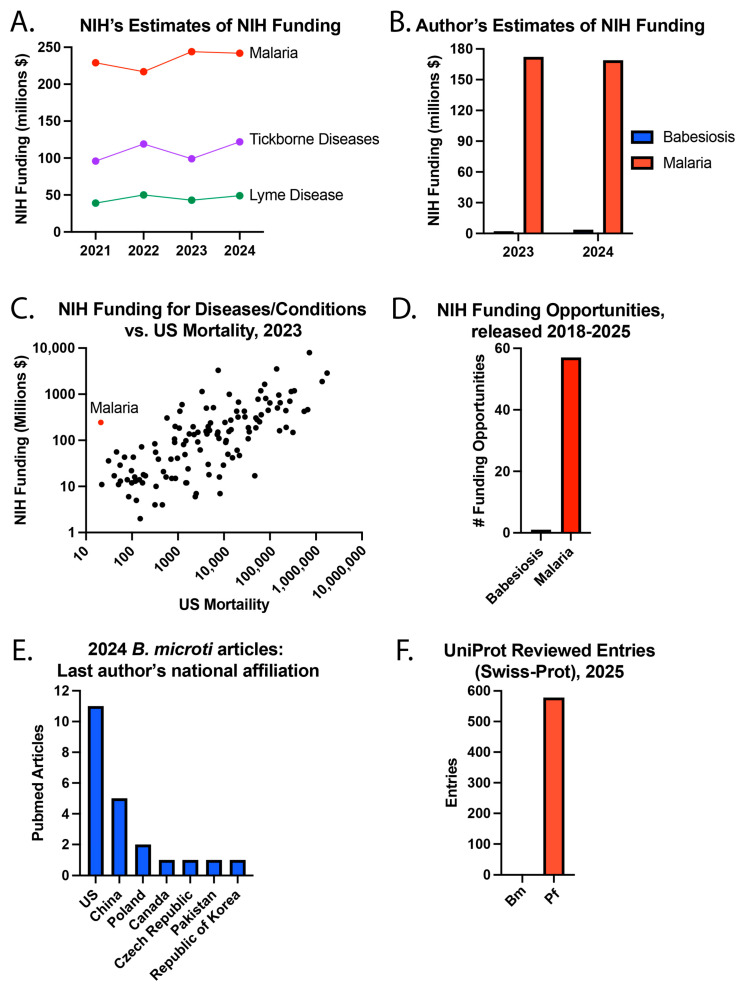
Babesiosis and malaria research. (**A**) NIH’s estimates of NIH funding, (**B**) the author’s estimates of NIH funding, (**C**) NIH funding for diseases/conditions compared to US mortality in 2023, (**D**) NIH funding opportunities released from 2018–25, (**E**) the national affiliation of the last author of PubMed articles about *Bm* published in 2024, and (**F**) UniProt reviewed entries in August 2025.

The NIH estimates NIH funding for diseases and conditions in the RCDC system. For example, $242 million of NIH funding was for malaria in 2024 (Figure 3), but babesiosis was not listed as a disease category. However, $122 million was for tickborne diseases and $49 million for Lyme disease, the most common tickborne disease in the US. Of note, NIH lists $73 million in malaria vaccine funding in 2024, and these funds are likely included in the total malaria funding. It is worth noting that NIH malaria funding has been estimated to be >$100 million for every year from 2008–2024, with a positive sloped trend line, which understandably and appropriately reflects the enormous global burden of malaria.

To estimate NIH funding for babesiosis and malaria, the NIH RePORTER website was used to search for NIH grants with the terms “*Babesia*” or “babesiosis” in the project title or “malaria”, “*Plasmodia*” or “*Plasmodium*” in the project title. Using this approach, fiscal year (FY) 2024 NIH funding (direct and indirect costs) was approximately $3.74 million for babesiosis and $169.21 million for malaria. A search for NIH funding opportunities [33] using the terms “malaria”, “*Plasmodium*” or “*Plasmodia*” led to 57 search results released from 2018-present, while there was 1 result for “babesiosis” or “*Babesia*” [33]. Of note, the NIH funding opportunities website was updated during the peer review process to remove Notices of Special Interest (NOSI) from the search results. 

To better understand NIH funding for diseases and conditions, the NIH’s estimate of NIH research funding was plotted against the number of US deaths in the year 2023. This year was selected because the mortality data is only listed for 2023 in the RCDC table. Some diseases did not have NIH funding and/or mortality estimates. Of the 120 diseases or conditions with complete data, malaria caused the fewest number of deaths (21) in 2023. The NIH provided $244 million in funding for malaria in 2023, which was higher than 81 (68.1%) of the other 119 diseases or conditions with complete data. These differences likely reflect important considerations regarding the global burden of disease. A separate example is that the NIH estimated $49 million in NIH funding for Zika virus in the year 2024, for which there hasn’t been a locally acquired case in a US state since 2017 [34]. Concurrently, the NIH estimated $15 million in funding for endemic West Nile Virus (WNV), the “leading domestically acquired arboviral disease” [35], in which an outbreak caused 1487 cases and 101 deaths in Maricopa County of Arizona in year 2021.

Given the global burden of malaria, the US has become a leading contributor to combating the disease worldwide, dedicating more resources than many other countries [36]. This commitment is exemplified through landmark initiatives such as the US President’s Malaria Initiative (PMI), which had played a pivotal role in reducing malaria cases and saving millions of lives. The US also provides approximately one-third of the funding for the Global Fund to fight AIDS, tuberculosis, and malaria. These commitments underscore the interconnected fight against devastating infectious diseases in general [37]. In FY2025, the US invested ~$1 billion on malaria [36], reinforcing its role as a global leader. In 2025, there were several changes made by the US federal government that impacted US malaria funding, such as the dissolution of the US Agency for International Development (USAID) [38].

Research and innovation have been especially transformative in driving progress against malaria. In 2023, malaria research and development totaled an estimated $690 million globally, with the NIH contributing $201 million and the Bill and Melinda Gates Foundation dedicating $181 million [26]. Breakthroughs funded by these investments have led to improved diagnostics, life-saving treatments, and effective prevention tools, offering hope to malaria-endemic regions.

While the global burden of babesiosis is substantially lower, it remains a significant concern for patients living in endemic regions. The narrower geographic distribution of infection may have inadvertently led to missed opportunities to apply advances from malaria research toward reducing the impact of babesiosis. Differences in awareness may have also been influenced by the contrasting geographic footprints of malaria and babesiosis outlined above. To gain insight into current babesiosis research in the US, a search was performed for articles published in 2024 about *Bm*, the most common cause of babesiosis in the US. Specifically, a search was performed for PubMed articles published in 2024 with “*Babesia microti*” in the title. A total of 22 articles were found, of which 11 (50%) of the last listed authors were from the US and 5 (22.7%) were from China.

Research funding is predicted to lead to medical progress. One objective approach to measuring our understanding of *Bm* and *Pf* parasites is by using UniProt, a comprehensive database of proteins [39]. In UniProt, there were 578 reviewed (Swiss-Prot) entries for *Pf* and 0 for *Bm* in August 2025. This suggests that little is known about *Bm* and much more about *Pf*, warranting a review of the history of these diseases.

### 3.5. History

Malaria is often listed as one of the all-time most common causes of human death and has dramatically impacted world history [40,41]. Malaria was present in the US in colonial times and may have helped the US gain independence from the British in the American Revolutionary War. Because many in the Continental Army and American militia previously had malaria from living in the South, they developed some immunity, while the British had not [40]. Malaria is thought to have impacted many other wars [41].

Our understanding of the *Plasmodium* parasite began around 1880, when Charles Louis Alphonse Laveran discovered that *Plasmodium* is the cause of malaria (1907 Nobel Prize in Physiology or Medicine). In 1897, Sir Ronald Ross showed that *Plasmodia* are transmitted to humans by mosquitoes (1902 Nobel Prize in Physiology or Medicine). In 1897, Johns Hopkins physician Dr. William H. Welch proposed the term *falciparum* to describe the deadliest *Plasmodium* species [42].

In the early 1900s, the US faced the challenge of malaria during the building of the Panama Canal, and with US troops located in the southern US and Cuba [43]. The US Public Health Service helped reduce malaria cases around the Tennessee River valley after President Franklin D. Roosevelt created the Tennessee Valley Authority as part of the New Deal. During World War II (WWII), the US created Malaria Control in War Areas (MCWA) to prevent US troops from contracting malaria in the southern US prior to deploying to war. In 1946, the Communicable Disease Center (CDC) arose from MCWA, with its major goal being the elimination of malaria in the US [43].

The National Malaria Eradication Program (NMEP) was a collaboration of the CDC with state and local health departments, created in 1947. By using DDT insecticide and removing mosquitoes’ breeding areas, malaria was eliminated from the US in 1951. The use of DDT as an insecticide was discovered about a decade earlier in 1939 by Paul Hermann Müller (1948 Nobel Prize in Physiology or Medicine) [44]. However, the environmental harm caused by DDT was highlighted in Rachel Carson’s 1962 book *Silent Spring* [45], which described the decline in bird populations, partly attributed to DDT causing birds to make thin eggshells. This book contributed to the environmental movement.

The first case of babesiosis was reported in 1966 in California, and the first case of *Bm* was reported in 1969 on Nantucket Island in Massachusetts [3]. Babesiosis cases in the US have increased for a variety of reasons, including greater awareness of this disease, increased human population, and climate change [46,47]. Consequently, the CDC made babesiosis a nationally notifiable disease in 2011. Although both *Bm* and *Borrelia burgdorferi*, which causes Lyme disease, are transmitted by the same tick species, cases of *Bm* have been more geographically restricted than Lyme disease. However, studies have recently reported *Bm* spreading into the mid-Atlantic region [48].

### 3.6. Medical Progress

Many medications have been discovered and developed to treat malaria over the course of centuries [49,50]. Notably, artemisinin was purified from *Artemisia annua* by Tu Youyou in 1971 (2015 Nobel Prize in Physiology or Medicine). Artemisinin-based combination therapies (ACTs) are commonly used in the treatment of malaria to reduce the likelihood of drug resistance. However, *Pfkelch13* mutations can cause artemisinin resistance. The CDC website currently states that *Pf* can demonstrate resistance to nearly all antimalarials [51]. Despite this, malaria chemoprophylaxis is highly effective.

In 1948, JBS Haldane’s malaria hypothesis helped explain that certain RBC variants, such as thalassemia trait, likely arose due to giving a survival advantage to individuals with malaria. Similarly, some surface antigen diversity on RBCs is thought to reflect evolutionary pressures imposed by *Plasmodium* [52,53,54,55,56,57]. For example, the highly prevalent blood group O is thought to have evolved from blood group A due to offering a survival advantage against *Pf* malaria, possibly due to decreased rosetting [54,58,59]. Karl Landsteiner discovered the ABO(H) blood groups in 1901 (1930 Nobel Prize in Physiology or Medicine). The diversity of RBC surface antigens necessitates comprehensive compatibility testing to ensure safe transfusion medicine practices, reduce the risk of alloimmunization in at-risk patients, and secure appropriate blood products [52,53,55,60]. Recent research has increasingly focused on the mechanisms driving antibody formation against RBC antigens and how these immune responses may impact clinical outcomes [61,62,63,64,65]. Studies have also examined the adverse consequences of transfusing antigen-incompatible blood and associated complications [66,67,68,69], particularly in the setting of highly alloimmunized patients [70,71,72], highlighting the complex intersection of patient immunology and transfusion practice in shaping clinical outcomes.

After malaria was eliminated from the US, researchers in and from the US continued to make key malaria discoveries. In 1975, the NIH reported that *P. knowlesi*, which typically causes malaria in Southeast Asia, was found to require Duffy (ACKR1) on RBCs for invasion [73]. This has therapeutic relevance because *Pk* malaria patients could be treated with RBC exchange transfusion, using Duffy-negative RBCs [74]. In 1976, the NIH found that the same RBC receptor was found to be important for *P. vivax* invasion [75]. In 1984, the NIH cloned and sequenced the immunodominant antigen on *Pf* sporozoites, known as circumsporozoite protein (CSP) [76]. Dr. Louis H. Miller, the current Chief of the Malaria Cell Biology Section at the National Institute of Allergy and Infectious Diseases (NIAID), contributed to these three major discoveries.

In the 1980s, GlaxoSmithKline and the Walter Reed Army Institute of Research created the RTS,S vaccine targeting the *Pf* CSP. Building on this achievement, the University of Oxford later developed an improved version, R21, which is manufactured at scale by the Serum Institute of India. *Pf* blood-stage vaccine research was facilitated by the development of continuous in vitro cultures of *Pf* established in 1976 at the Rockefeller University in NY [77]. The leading *Pf* blood-stage vaccine candidates are targeted against *Pf*Rh5 and *Pf*CyRPA ligands, which bind to basigin and glycans on RBCs, respectively [78].

Regarding babesiosis, all medications used to treat *Bm* (e.g., atovaquone, azithromycin, clindamycin, quinine) were developed for other purposes [79]. There is neither chemoprophylaxis nor a vaccine for human babesiosis. By knowing key *Bm* ligand and host cell receptor interactions, it is possible that a vaccine or invasion-blocking medications could be developed [80]. While there have been many *Pf* ligand and RBC receptor pairs identified [81], none are known for *Bm*. This is probably because the first description of continuous in vitro cultures of *Bm* was published in 2024 (Table 1) [82]. This technological limitation likely delayed progress in identifying pairs of *Bm* ligands and RBC receptors; however, low levels of research funding slow the rate at which technological limitations can be overcome. It is conceivable that *Bm* invasion-resistant RBCs could be used in an RBC exchange transfusion to treat patients, as has been tried for *B. divergens* [83,84], the most common cause of babesiosis in Europe.

## 4. Discussion

This study found that over a 5-year period, there were more babesiosis cases and deaths in the US compared to malaria. Yet, US public health efforts, medical interventions, and US research funding are more robust for malaria than for babesiosis. The US made great advancements against malaria by providing decades of robust funding to organizations such as the CDC and NIH, and a similar strategy would surely work for babesiosis.

In 2009, before babesiosis was added to the list of nationally notifiable diseases, Dr. Peter Krause, a leading babesiosis expert at Yale School of Public Health, noted that “Babesiosis has gotten little respect and very little funding,” emphasizing that “It’s far more important than people realize” [87]. Many physicians and researchers may simply have limited awareness of the disease because it is geographically restricted. Similar barriers have been suggested for conditions such as sickle cell disease and alpha-gal syndrome [69,88,89], which also struggle to attract broad attention. This lack of visibility makes it more difficult to secure nationwide attention from lawmakers and funding agencies, not due to intentional neglect, but because other pressing health concerns understandably often take precedence. Increased attention on babesiosis could lead to better prevention and treatment options.

There were ~1960 confirmed babesiosis cases annually in the US. Because the average life expectancy in the US is 78.4 years [90], there are probably <154,000 living persons in the US who have ever had babesiosis. The Cleveland Clinic defines a “rare disease” as impacting <200,000 persons in the US, while an “orphan disease” is one for which there is little research [91]. Our study found there is ~$4 million in annual NIH funding for babesiosis. A search of PubMed for “babesiosis” AND “orphan disease” yielded zero results, while a search of “amyotrophic lateral sclerosis” AND “orphan disease” yielded more than a dozen. Although we have not seen babesiosis described as an orphan disease, our findings suggest that it is and should be eligible for orphan disease research funding.

There is still much to learn about *Bm* and valuable insights can be drawn from the extensive body of research on *Pf*. For example, RBC traits such as surface antigen expression, hemoglobin variants, and enzymatic deficiencies have been well documented as key factors influencing *Plasmodium* infection risk and disease severity, yet similar studies for *Bm* are scarce [85,92]. By knowing which *Bm* ligands and RBC receptors are important for invasion, therapeutic approaches and vaccines can be developed.

As with all reports, this study has several limitations. This study primarily examined the US. Globally, malaria remains one of the most devastating infectious diseases, with the World Health Organization reporting 263 million cases and 597,000 deaths globally in 2023, disproportionately affecting developing countries and particularly vulnerable populations such as young children and pregnant women in sub-Saharan Africa [26]. Despite reductions achieved through vector control, community engagement, vaccines, and novel interventions, some estimates have suggested that progress on malaria control progress has plateaued, demonstrating that research and innovation continue to be urgently needed. Monoclonal antibodies to prevent *Pf* malaria are being developed, and gene drive technology may be used to hinder the ability of *Anopheles* mosquitoes to transmit *Plasmodium* parasites [93,94,95]. Another limitation is that this study focused on NIH and US-based funding for babesiosis and malaria. Because malaria poses a major global health burden, both endemic and non-endemic countries have contributed substantially to related research.

There are additional limitations. CDC’s definition of confirmed babesiosis or malaria can rely on microscopy, but *Babesia* and *Plasmodium* parasites can be difficult to distinguish based on morphology. A history of recent travel, among other details, can help distinguish the diseases. The number of US cases of babesiosis and malaria may not include individuals who did not seek medical care. Because the vast majority of malaria cases are imported, pandemic-related travel restrictions likely caused a greater decline in cases of malaria than babesiosis. Consistent with this, there was a much larger decrease in cases of malaria than babesiosis in 2020. Before the COVID-19 pandemic (in 2018 and 2019), there were more confirmed cases of babesiosis than malaria. Our estimates of NIH funding, funding opportunities, and published *Bm* articles could have missed relevant results that were not detected with the search terms selected. For example, in 2022, the NIH released a Notice of Special Interest for Advancing Research for Tickborne Diseases. Although the terms babesiosis or *Babesia* were not used in the document, it was likely relevant to babesiosis. These limitations could impact both babesiosis and malaria results.

Climate change is one factor that could impact the future numbers of US babesiosis and malaria cases. Because babesiosis cases are dependent on the tick season, mammalian hosts, climate, and other factors, there can be substantial fluctuations in these numbers annually [96]. Similarly, the habitat of *Anopheles* mosquitoes, which transmit *Plasmodium* parasites, is expanding due to climate change [97]. Ultimately, it seems prudent to study infectious diseases before they become bigger problems.

In conclusion, while babesiosis cases and deaths exceed those of malaria in the US, babesiosis lags malaria regarding public health efforts, research funding, and medical progress. Data suggest that babesiosis is both a rare disease and an orphan disease in the US, warranting greater CDC and NIH funding. Many unanswered questions remain that hinder a deeper understanding of the disease and limit advances in prevention and treatment. Adapting proven, malaria-related strategies to babesiosis could lead to greater prevention and medical care for this disease. Because the vast majority of babesiosis cases occur in the US, the US must take the lead in conquering this infectious disease.

## Figures and Tables

**Table 1:** Comparison of *Bm* babesiosis and *Pf* malaria.

	*Bm* Babesiosis	*Pf* Malaria
Endemic in US	Yes (and spreading geographically)	No (eliminated in 1951)
1st human case acquired & reported in the US	1969	Colonial times
High risk for severe disease	Elderly, asplenic, immunocompromised	Children, those with certain blood types
Most human cases in the world	US	Nigeria [26]
Foreign significance	As many as ~300 symptomatic human cases in China [1]	Hundreds of millions of cases globally and hundreds of thousands of deaths
Key mammalian hosts	Mice	Humans
Vectors (definitive hosts)	Ticks	Mosquitoes
Parasite stages in humans	Erythrocytic	Hepatic & Erythrocytic
Free CDC testing after diagnosis	No	Yes
Led to drug development & approval	No	Yes (e.g., quinine, chloroquine, artesunate, artemether, etc.)
Drug resistant strains	Yes	Yes
Chemoprophylaxis for humans	No	Yes
Vaccines	None for humans	RTS,S & R21
Blood donor precautions	*Babesia* NAT testing	Foreign travel & residence deferrals
Impact of RBC antigens on disease	Very few studies [85]	Extensively studied
UniProt reviewed entries (Swiss-Prot)	0 entries	578 entries
Parasite ligand & RBC receptor pairs	No pairs identified	Many pairs identified (e.g., *Pf*Rh5 and basigin)
Continuous in vitro cultures in human RBCs	Published in 2024 [82]	Published in 1976 [77]
Small animal models of disease	*B. microti* naturally infects mice	*Pf* can infect humanized mice [86]

## Data Availability

Data derived from public domain resources.
